# Miscellaneous Notices

**Published:** 1842-10-01

**Authors:** 


MISCELLANEOUS NOTICES.

***Mesmerism at Richmond, Va.*** Considerable stir having been recently made at Richmond, Va., by a course of alleged experiments on mesmerism, performed by a Mr. French, several of the physicians of the city undertook, by request, to investigate the subject. Their examinations resulted in the adoption of the following resolution:

*Resolved*, That after a careful examination of the experiments on Mesmerism, conducted by them at the Exchange Hotel, on the 16th of September, on Mr. William Ritter's girl Emma, and those previously performed on Mr. Pepper, at the same place, on the 6th instant, they are *decidedly* of the opinion that they do not tend in the *least degree* to establish its claims.

*Professor of Chemistry in the University of Virginia.* Dr. Robert E. Rogers, of Philadelphia, has been appointed to the Chair of Chemistry and Materia Medica, in the University of Virginia, vacated by the death of Dr. Emmet. Dr. Rogers officiated, during the last session, as Professor, and gave general satisfaction.

*Edinburgh University.* Dr. Home has resigned the Professorship of Practical Medicine, and has been succeeded by Dr. Alison, who vacates the chair he held—that of the Theory or Institutes of Physic. Dr. Home for many years held the latter professorship; and his successor in either chair is to relinquish £150, so as to make up a retiring allowance of £300 per annum.

Dr. John Thomson has also resigned his chair—that of Pathology; and Dr. Henderson has been elected his successor.

The vacancy in the Chair of Surgery, caused by the death of Sir Charles Bell, has been supplied by the appointment of Mr. Miller, one of the Surgeons to the Edinburgh Infirmary.

*Death of Pelletier.* This distinguished Chemist, Professor in the School of Pharmacy, died in the month of July last, after a long and painful illness. His researches on the vegetable alkalies, in association with M. Caventou, were a valuable contribution to science.

The post of Inspector General and member of the "Conseil de Santé des armées," filled by Baron Larrey, has been given to M. Begin, first Professor at the Val-de-Grâce.

